# Using the Internet to Promote Health Behavior Change: A Systematic Review and Meta-analysis of the Impact of Theoretical Basis, Use of Behavior Change Techniques, and Mode of Delivery on Efficacy

**DOI:** 10.2196/jmir.1376

**Published:** 2010-02-17

**Authors:** Thomas L Webb, Judith Joseph, Lucy Yardley, Susan Michie

**Affiliations:** ^3^Research Department of ClinicalEducational & Health PsychologyUniversity College London1-19 Torrington PlaceLondonUK; ^2^School of PsychologyShackleton BuildingUniversity of SouthamptonHighfieldSouthamptonUK; ^1^Department of PsychologyUniversity of SheffieldWestern BankSheffieldUK

**Keywords:** Internet, intervention, behavior change, meta-analysis, review

## Abstract

**Background:**

The Internet is increasingly used as a medium for the delivery of interventions designed to promote health behavior change. However, reviews of these interventions to date have not systematically identified intervention characteristics and linked these to effectiveness.

**Objectives:**

The present review sought to capitalize on recently published coding frames for assessing use of theory and behavior change techniques to investigate which characteristics of Internet-based interventions best promote health behavior change. In addition, we wanted to develop a novel coding scheme for assessing mode of delivery in Internet-based interventions and also to link different modes to effect sizes.

**Methods:**

We conducted a computerized search of the databases indexed by ISI Web of Knowledge (including BIOSIS Previews and Medline) between 2000 and 2008. Studies were included if (1) the primary components of the intervention were delivered via the Internet, (2) participants were randomly assigned to conditions, and (3) a measure of behavior related to health was taken after the intervention.

**Results:**

We found 85 studies that satisfied the inclusion criteria, providing a total sample size of 43,236 participants. On average, interventions had a statistically small but significant effect on health-related behavior (d_+_ = 0.16, 95% CI 0.09 to 0.23). More extensive use of theory was associated with increases in effect size (*P* = .049), and, in particular, interventions based on the theory of planned behavior tended to have substantial effects on behavior (d_+_ = 0.36, 95% CI 0.15 to 0.56). Interventions that incorporated more behavior change techniques also tended to have larger effects compared to interventions that incorporated fewer techniques (*P* < .001). Finally, the effectiveness of Internet-based interventions was enhanced by the use of additional methods of communicating with participants, especially the use of short message service (SMS), or text, messages.

**Conclusions:**

The review provides a framework for the development of a science of Internet-based interventions, and our findings provide a rationale for investing in more intensive theory-based interventions that incorporate multiple behavior change techniques and modes of delivery.

## Introduction

*...without a scientific underpinning, the field [of Internet interventions] may flounder [1] *[LM Ritterband and DF Tate]

In June 2009 an estimated 25% of the world’s population had access to the Internet, with estimates in Europe and North America being considerably higher (50% and 74%, respectively) [2]. Researchers in the field of health promotion have been quick to capitalize on the exponential growth of the Internet, and over the past decade, an increasing number of interventions designed to promote changes in health behavior have been delivered via the Internet [1,3]. For example, “Happy Ending” is a 54-week Internet-based intervention designed to promote smoking abstinence [4,5]. This intervention involves over 400 contact emails that direct participants to a different webpage each day, supplemented by interactive voice response (IVR) and short message service (SMS) monitoring and prompts. Other Internet-based interventions, however, simply involve embedding a short planning exercise within an online lifestyle survey [6,7].

Quantitative reviews of Internet-based interventions report positive–albeit highly variable and often small–effects on behaviors such as physical activity, tobacco use, exercise, and so on [8-12]. However, previous reviews have not systematically coded the characteristics of each Internet-based intervention and computed the effect size associated with each [1]. The limited analyses of this kind that have been conducted suggest that this approach may provide insight into the characteristics of effective versus less effective interventions. For example, Portnoy et al [8] coded whether the intervention included information, motivation, or behavioral skill components. The findings suggested that the inclusion of motivational components (eg, cost-benefit analyses) actually weakened the impact of the interventions. Since the publication of the review by Portnoy et al [8], a comprehensive taxonomy of behavior change techniques has been published [13], along with a method for assessing the extent to which behavioral interventions are theory-based [14]; both these developments permit more sophisticated coding of intervention content. Thus, the primary aim of the present review was to use these new coding schemes to identify the characteristics of effective Internet-based interventions. A secondary aim was to develop a coding scheme for the different modes by which Internet-based interventions are delivered (eg, via scheduled access to an advisor or automated feedback) and to link different modes of delivery to effect size.

### How Can the Characteristics of Internet-based Interventions Be Conceptualized?

Three intervention characteristics may influence the impact on behavior [15-18]: (1) the theoretical basis of the intervention, (2) the behavior change techniques used, and (3) the mode of delivery.

#### Theoretical Basis and Use of Theory and Predictors

Theoretical basis refers to the theory or theories used to develop the intervention. For example, in an effort to promote physical activity, Spittaels et al [19] directed participants to a website that presented a tailored message based on the theory of planned behavior [20]. In contrast, Carr et al [21] used social cognitive theory [22] to develop a physical activity intervention that could be delivered via the Internet. Theory can inform interventions in a number of different ways, from identifying theoretical constructs to be targeted (eg, attitude, self-efficacy) or mechanisms underlying particular behavior change techniques (eg, vicarious learning in modeling), to selecting participants most likely to benefit (eg, people with particularly negative attitudes). Despite assertions that use of theory leads to more effective interventions [23-27], there is debate over the importance of theory [28,29], and at present it is unclear whether and how use of theory influences intervention effectiveness, particularly in relation to Internet-based interventions [1]. A large review of HIV-prevention interventions reported that use of theory was positively related to extent of behavior change [30], but this finding was simply based on whether or not theory was cited. Although this is an important step in the right direction, it would be useful to know how different uses of theory impact on the effectiveness of interventions and whether more extensive use of theory leads to larger effects than less extensive use. Michie and Prestwich [14] have developed a reliable coding scheme to assess the different ways that behavioral interventions employ theory; use of this coding scheme permits the present review to investigate these important questions.

#### Behavior Change Techniques

Behavior change techniques refer to the specific strategies used in the intervention to promote behavior change. For example, some interventions designed to promote smoking abstinence prompt barrier identification and problem solving (eg, [31]), whereas other interventions prompt participants to monitor their behavior (eg, [32]). In order to identify techniques contributing to effectiveness across interventions and to ensure that effective interventions can be replicated, it is crucial that standardized definitions of the techniques included in behavior change interventions are used and linked to intervention effectiveness [33]. With this in mind, the present review used the taxonomy of behavior change techniques developed by Abraham and Michie [13] to code the content of the interventions.

#### Mode of Delivery

The interventions in the present review were delivered via the Internet. The effects of this primary mode of delivery can be estimated by examining studies that compare similar materials presented via the Internet versus other modes, such as print [34,35]. Internet-based interventions can, however, differ substantially in their specific mode of delivery. For example, content can be delivered in a more or less interactive manner [36,37]. Interventions may also employ supplementary delivery modes (eg, SMS messaging, email, telephone, or videoconferencing) that may influence effectiveness. To our knowledge, no coding scheme exists for assessing the mode with which Internet-based interventions are delivered. Existing coding schemes developed for systematic reviews of non-Internet interventions [38] are not suitable because they focus on the physical manner in which participants received the intervention (eg, one-to-one or group) and the nature of the person delivering the intervention (eg, health educator or trained facilitator). Therefore, the present review developed a new coding scheme for assessing mode of delivery in Internet-based interventions and used it to understand how each mode influences the effectiveness of the intervention.

### The Present Review

The present review sought to investigate which characteristics of Internet-based interventions were associated with effectiveness. By so doing, we answer the important applied and theoretical questions: Which theories should researchers draw on in developing interventions? How can theory best be used to inform Internet-based interventions? What behavior change techniques are effective when employed via the Internet? Is the mode by which the intervention is delivered important?

## Method

### Selection of Studies

#### Identification and Screening

In July 2008 we conducted a computerized search using ISI Web of Knowledge, which covers a number of databases including Web of Science conference proceedings (1900-), BIOSIS Previews (1985-), and Medline (1950-). We used the following search terms: Web-based, Internet, digital, online, technolog*, computer, treatment, RCT, trial, intervention, behavio* change. (The asterisk automatically truncates the term such that, for example, technolog* will also find technology, technologies, etc). Studies had to include one or more of the search terms in the title. We also sent an email to the distribution list of the European Health Psychology Society to request unpublished research. There were three inclusion criteria for the meta-analysis. First, the primary components of the intervention must have been delivered via the Internet (not including CD-ROMs, SMS messaging, or other computer applications). Second, the studies must have involved random assignment of participants to a treatment group that received an Internet-based intervention and a comparison group that received either a control intervention or no intervention. Finally, a measure of behavior related to health must have been taken after the intervention. We did not include studies that only measured symptoms (eg, anxiety, depression), cognitions (eg, attitudes, intentions), outcomes presumed to be the consequence of behavioral changes (eg, weight loss, blood glucose levels), or behaviors unrelated to health (eg, use of literature services).

#### Eligibility and Inclusion

**Figure 1:**
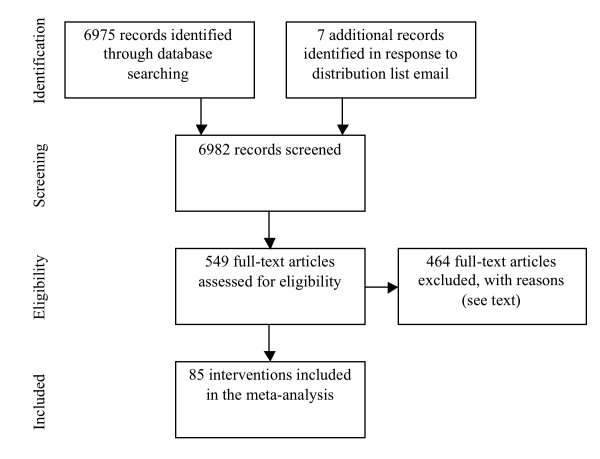
Flow of information through the different phases of the review (adapted from [48])

Figure 1 shows the flow of information through the different phases of the review. We assessed 549 full-text articles for eligibility. Of these, 140 studies (26%) were rejected because the study did not include a measure of behavior related to health (eg, [39]), 97 studies (18%) were rejected because the primary components of the intervention were not delivered via the Internet (eg, [40]), 88 studies (16%) were rejected because they did not report intervention effects (typically, these were reviews or protocol descriptions, eg, [41]), 84 studies (15%) were rejected because they did not include a control group (eg, [42]), 20 studies (4%) were rejected because computers were used only to tailor information that was presented in a non-computerized format (eg, [43]), 17 studies (3%) were rejected because they reported additional effects of an intervention already included in the review (eg, [44]), 8 studies (1%) were rejected because intervention effects were reported in a manner that did not permit computation of an effect size (eg, [45]). For these studies, it was decided not to estimate effect sizes based on the significance levels reported because all the effects for which full information was not available were reported as non-significant. Assuming zero difference (*d * = 0.00) for these effects could systematically underestimate effect sizes associated with particular intervention characteristics. Finally, 5 studies (1%) were rejected because participants were not randomly allocated to conditions (eg, [46]), and 4 studies (1%) were rejected because the manuscripts were not written in English (eg, [47]). In total, 85 reports of Internet-based interventions met the inclusion criteria for the review. Multimedia Appendix 1 presents the characteristics and effect sizes associated with each intervention.

### Calculation of Effect Sizes for the Effect of Internet-Based Interventions on Health-Related Behavior

The effect size for post-intervention behavior differences between the conditions was calculated in line with Cochrane recommendations [49]. Specifically, the longest follow-up was selected wherever possible. For example, where Brendryen et al [5] followed up smokers at 3, 6, and 12 months, the 12-month data was included in the review. Where studies examined more than one behavior (such as Williamson et al’s study [50] of weight loss behaviors in which exercise, overeating, and avoidance of fattening foods were measured), the effect sizes within the study associated with different behaviors were meta-analysed in their own right prior to inclusion in the main dataset. This procedure captures the richness of the data and does not prioritize one outcome over another (eg, effects on dietary outcomes and effects on physical activity are considered equally important), while also maintaining the independence of samples that is central to the validity of meta-analysis [51]. Intention-to-treat analyses were used wherever possible. Following Portnoy et al [8], where studies employed more than one comparison condition, we selected the most passive comparison condition for ease of interpretation. For a detailed discussion of considerations relating to choice of comparison conditions, see Danahar and Seeley [53].

### Coding of Intervention Characteristics

#### Use of Theory and Predictors

The coding scheme developed by Michie and Prestwich [14] was used to code how theory and predictors (constructs that are not explicitly linked to a theory by the authors but are targeted for intervention because they predict behavior) were used in the design of the interventions. Items 1 through 6 of the coding scheme identify whether theory or predictors are mentioned and whether they are used to select recipients for the intervention, to select or develop intervention techniques, or to tailor intervention techniques to participants. Items 7 through 11 examine whether intervention techniques are explicitly linked to theory-relevant constructs or predictors and, conversely, whether theory-relevant constructs or predictors are linked to intervention techniques. Items 12 through 17 were not evaluated in the present review because they do not pertain to use of theory in developing the intervention. These items focus on methodological issues (randomization and measurement quality) and whether theory was refined on the basis of outcomes. Where the theoretical basis of the experimental intervention was identical to that of the comparison intervention (eg, [34]), the intervention was coded as not having a theoretical basis that could explain differences in effect size between the conditions.

In addition to considering each use of theory separately, we also summed items 1 through 11 to create an overall “use of theory” score that could be used to evaluate whether more extensive use of theory leads to larger effects than less extensive use. In a slight change to the published recommendations, item 8 (“At least one, but not all, intervention techniques are explicitly linked to at least one theory-relevant construct/predictor”) was coded as “yes” if item 7 (“All intervention techniques are explicitly linked to at least one theory-relevant construct/predictor”) was coded as “yes.” Similarly, item 11 (“At least one, but not all, theory-relevant constructs/predictors are explicitly linked to at least one intervention technique”) was coded as “yes” if item 10 (“All theory-relevant constructs/predictors are explicitly linked to at least one intervention technique”) was coded as “yes.” This ensured that when we created the ”use of theory” score, reports that linked, for example, all theoretical constructs with intervention techniques, were also credited as linking some theoretical constructs with intervention techniques.

#### Theoretical Basis

Interventions were coded as having a particular theoretical basis only if the theory was used to develop the intervention techniques (item 5 of the coding scheme of Michie and Prestwich [14]) rather than theory being simply mentioned (item 1).

#### Behavior Change Techniques

The behavior change techniques used in the interventions were coded using an augmented 40-item version [52] of the 26-item taxonomy developed by Abraham and Michie [13] (see Table 2 for a list of techniques). Where the behavior change techniques used by the experimental intervention were the same as those in the comparison intervention (eg, [35]), the experimental intervention was coded as not using any behavior change techniques.

#### Mode of Delivery

Mode of delivery was coded using a novel coding scheme developed by the present authors. For convenience, we divided mode of delivery into (i) automated functions, (ii) communicative functions, and (iii) use of supplementary modes. Each category included a list of delivery modes, and we marked whether or not each intervention used that mode. Automated functions included: (a) the use of an enriched information environment (eg, supplementary content and links, testimonials, videos, or games), (b) automated tailored feedback based on individual progress monitoring (eg, comparison to norms or goals, reinforcing messages, or coping messages), and (c) automated follow-up messages (eg, reminders, tips, newsletters, encouragement). Communicative functions included: (d) access to an advisor to request advice (eg, “ask the expert” facility, expert-led discussion board, or chat sessions), (e) scheduled contact with advisor (eg, emails), and (f) peer-to-peer access (eg, buddy systems, peer-to-peer discussions boards, forums, or live chat). Finally, use of supplementary modes included the use of (g) email, (h) telephone, (i) Short Messaging Service (SMS), (j) CD-ROM, or (k) videoconferencing.

The features of intervention delivery that we coded were, to a large extent, constrained by the features that authors typically report and that can be easily and objectively verified (eg, whether text messages were used). The list is not intended to be exhaustive and we recognize that there are other features that may be important but that are not routinely used or reported, or that are hard to measure. For example, navigational format (eg, the extent to which users are “tunnelled” to particular information vs given free choice [54]), entertainment value (eg, use of quizzes, stories, graphics), appearance (eg, color, layout, screen size [18]), and credibility (eg, the extent to which the website cites sources, credentials). As Internet-based interventions become more common and standards of reporting improve, it should be relatively easy to integrate these additional delivery features into the present coding scheme.

### Meta-analytic Strategy

We used Hedges *g* as the primary estimate of effect size for each intervention. Hedges *g* is the difference between the two means (for experimental and control conditions, respectively) divided by the pooled standard deviation. Computations were undertaken using Comprehensive Meta-Analysis Version 2 (Biostat, Englewood, NJ, USA) [55] with the exception of meta-regression computations for which we used the weighted least squares regression command in SPSS 15 for Windows (SPSS Inc, Chicago, IL, USA). Weighted average effect sizes (d_+_) were based on a random effects model because studies were likely to be “different from one another in ways too complex to capture by a few simple study characteristics” [56]. Effect sizes were interpreted using Cohen’s [57] guidelines. According to Cohen, d_+_= .20 should be considered a “small” effect size, d_+_= .50 is a “medium” effect size, whereas d_+_= .80 is a “large” effect size. The homogeneity Q statistic [58] was used to evaluate variability across effect sizes from the primary studies. When Q is statistically significant it indicates that the effect sizes are heterogeneous. For the meta-regressions, ß is beta weight or coefficient assigned to the predictor and *t *(and the associated *P *-value) tests whether the beta weight is significantly different from zero.

## Results

### Effect of Internet-based Interventions on Health-related Behavior

The weighted average effect size across all interventions was d_+_= 0.16 with a 95% confidence interval from 0.09 to 0.23 based on 85 studies (k = 85) and a total of 43,236 participants (see Table 1). This means that the Internet-based interventions had, on average, a small effect on health behavior according to Cohen’s criteria [57]. While these qualitative indices are useful for interpreting the findings of systematic reviews, however, statistical effectiveness is not necessarily the same as clinical effectiveness. For example, a relatively small effect of an Internet-based intervention on smoking abstinence could have substantial clinical significance [59]. On the other hand, an Internet-based intervention that produces a reliable change in fat intake has the potential to benefit a larger proportion of the population than an intervention targeted at smokers. Given that much of the cost associated with Internet-based interventions is likely to be incurred at the design and development stage rather than in delivering individual treatments, small effects with the potential to have an impact on large numbers of people may thus be significant for patient or population health.

We also calculated effect sizes separately for commonly targeted behaviors (see Table 1). Small, but significant, effects on behavior were observed for Internet-based interventions that targeted only physical activity (d_+_= 0.24, k = 20, 95% CI 0.09 to 0.38), dietary behavior (d_+_= 0.20, k = 10, 95% CI 0.02 to 0.37), or alcohol consumption (d_+_= 0.14, k = 9, 95% CI 0.00 to 0.27). Interventions that targeted smoking abstinence tended to have slightly smaller effects on behavior that did not reach statistical significance (d_+_= 0.07, k = 12, 95% CI -0.04 to 0.18). Finally, we calculated effect sizes separately for interventions that targeted multiple behaviors (eg, Williamson et al’s intervention [50] targeted physical activity and dietary behavior) and those that targeted a single behavior. Interventions that targeted multiple behaviors tended to have slightly smaller effects on behavior (d_+_= 0.12, k = 10, 95% CI 0.08 to 0.17) than did interventions that targeted a single behavior (d_+_= 0.17, k = 75, 95% CI 0.09 to 0.24), although both effects were statistically significant.

**Table 1:** Weighted effect sizes (d+) for behavior change as a function of Internet-based interventions by behavior type

Behavior	*k * ^a^	*Q * ^b^	95% CI	d_+_^c^
Physical activity	20	128.76^f^	0.09-0.38	0.24^e^
Dietary behavior	10	30.82^e^	0.02-0.37	0.20^f^
Alcohol consumption	9	47.45^f^	0.00-0.27	0.14^d^
Smoking abstinence	12	45.46^e^	-0.04 to 0.18	0.07
Interventions targeting multiple behaviors	10	7.90	0.08-0.17	0.12^f^
Interventions targeting a single behavior	75	879.81^f^	0.09-0.24	0.17^f^
All studies	85	896.67^f^	0.09-0.23	0.16^f^

^a^k = the number of interventions included in the estimate of effect size

^b^Q = homogeneity for the subgroup of interventions

^c^d+ = weighted average effect size

^d^ *P *< .05

^e^ *P *< .01

^f^ *P *< .001

### Intervention Characteristics

Across all interventions, the homogeneity Q statistic was highly significant (Q = 896.67, *P *< .001), which indicates considerable variability across effect sizes from the primary studies. To examine the impact of intervention characteristics on effect size, we computed the weighted average effect size for behavior change as a function of the theoretical basis of the interventions, the different ways that the interventions used theory, the behavior change techniques, and the mode of delivery. The findings from these analyses are shown in Table 2. Multimedia Appendix 2 shows the characteristics of each intervention.

#### Use of Theory and Predictors

Of the different uses of theory proposed by Michie and Prestwich’s coding scheme [14], theory or predictors were most commonly used to select or develop intervention techniques (k = 37). Over 20% of the interventions, however, mentioned theory (k = 30), linked at least one intervention technique to theory (k = 19), linked at least one theory-relevant construct to an intervention technique (k = 18), or mentioned a target construct as a predictor of behavior (k = 18). Interventions that used theory or predictors to select recipients for the intervention tended to have the largest effects on behavior (d_+_= 0.33, k = 3, 95% CI 0.15 to 0.52) with most other uses of theory tending to have smaller effects (Median d_+_= 0.19). Overall, meta-regression indicated that increased use of theory had a significant positive impact on effect sizes (ß = 0.22, *t *= 2.00, *P *= .049). Interventions that made extensive use of theory tended to have larger effects on behavior than did interventions that made less extensive or no use of theory.

#### Theoretical Basis

Only three theories were used by three or more studies to develop the intervention; social cognitive theory (SCT) [22], the transtheoretical model (TTM) [60], and the theory of reasoned action/planned behavior (TPB) [20,61]. Effect sizes associated with interventions based on the TPB tended to have larger effects on behavior (d_+_= 0.36, k = 9, 95% CI 0.15 to 0.56) than did interventions based on the TTM (d_+_= 0.20, k = 12, 95% CI 0.08 to 0.33) that, in turn, had larger effects than did interventions based on SCT (d_+_= 0.15, k = 12, 95% CI 0.04 to 0.25).

#### Behavior Change Techniques

The most commonly used behavior change techniques (used by 30% or more of interventions) were providing information on the consequences of behavior in general (k = 29), prompting self-monitoring of behavior (k = 28), and identifying barriers and/or problem solving (k = 26). The largest effects on behavior were observed for interventions that provided stress management (d_+_= 0.50, 95% CI 0.27 to 0.72) or general communication skills training (d_+_= 0.49, 95% CI 0.25 to 0.73), although these were used by relatively few interventions (k = 5 and 3, respectively). Modeling, relapse prevention/coping planning, facilitating social comparison, goal setting, action planning, and provision of feedback on performance all had effects on behavior that exceeded d_+_= 0.20 (Median d_+_= 0.28). Finally, a few strategies had small and non-significant effects on behavior: use of follow-up prompts, self-monitoring of behavioral outcome, emotional control training, and provision of information about others approval. Overall, meta-regression indicated that the number of behavior change techniques employed had a significant positive impact on effect size (ß = 0.36, *t *= 3.48, *P *< .001). Interventions that used more techniques tended to have larger effects on behavior than did interventions that used fewer techniques.

#### Mode of Delivery

Only one mode of delivery was used by 30% or more of interventions–providing an enriched information environment (k = 30). Over 20% of interventions, however, provided access to an advisor to request advice (k = 23), used peer-to-peer access (k = 20), used email in addition to the Internet-based intervention (k = 19), or provided automated tailored feedback (k = 18). For convenience of interpretation, effect sizes for modes of delivery were divided into three subgroups: automated functions, communicative functions, and use of supplementary modes. In terms of automated functions, small, but significant, effects on behavior were observed for interventions that provided automated tailored feedback (d_+_= 0.18, k = 18, 95% CI 0.07 to 0.28) or an enriched information environment (d_+_= 0.15, k = 30, 95% CI 0.07 to 0.23). Interventions that provided automated follow-up messages tended not to have significant effects on behavior (d_+_= 0.09, k = 14, 95% CI -0.01 to 0.19). Of the communicative functions, interventions that provided access to an advisor to request advice tended to have small-to-medium effects on behavior (d_+_= 0.29, k = 23, 95% CI 0.16 to 0.42), while smaller effects on behavior were observed for interventions that provided scheduled contact with an advisor (d_+_= 0.22, k = 13, 95% CI 0.09 to 0.36) or peer-to-peer access (d_+_= 0.20, k = 20, 95% CI 0.09 to 0.21). Finally, use of additional modes appeared to have distinct effects on behavior change with Internet-based interventions that also used text messages having large effects on behavior (d_+_= 0.81, k = 4, 95% CI 0.14 to 1.49), Internet-based interventions using the telephone having small-to-medium effects (d_+_= 0.35, k = 7, 95% CI 0.09 to 0.61), and interventions using email as an additional mode of delivery tending to have small effects on behavior (d_+_= 0.18, k = 19, 95% CI 0.07 to 0.29).

**Table 2:** Effect sizes^a^ by theoretical basis, use of theory, behavior change techniques, and mode of delivery. The numbering for use of theory, behaviour change techniques, and the letters for mode of delivery correspond with those items in the coding frames and Multimedia Appendix 2.

		K^b^	Q^c^	95% CI	d_+_^d^

**Theoretical Basis **					
	Theory of reasoned action/planned behavior (TPB) [20,61]	9	108.44^h^	0.15 to 0.56	0.36^g^
	Transtheoretical model (TTM) [60]	12	68.99^h^	0.08 to 0.33	0.20^g^
	Social cognitive theory (SCT) [22]	12	18.62	0.04 to 0.25	0.15^g^
	Elaboration likelihood model (ELM) [62]	2			
	Extended parallel process model (EPPM) [63]	1			
	Self-regulation theory (SRT) [64]	1			
	Precaution adoption process model (PAPM) [65]	1			
	Diffusion of innovations model (DIM) [66]	1			
	Health belief model (HBM) [67,68]	1			
	Social norms theory (SNT) [69]	1			

**Use of Theory **					
	4. Theory/predictors used to select recipients for the intervention	3	2.84	0.15 to 0.52	0.33^h^
	9. Group of techniques are linked to a group of constructs/predictors	6	9.85	0.03 to 0.43	0.23^f^
	5. Theory/predictors used to select/develop intervention techniques	37	191.40^h^	0.13 to 0.29	0.21^h^
	2. Targeted construct mentioned as predictor of behavior	18	60.07^h^	0.11 to 0.31	0.21^g^
	6. Theory/predictors used to tailor intervention techniques to recipients	11	67.75^h^	0.07 to 0.34	0.21^g^
	1. Theory/model of behavior mentioned	30	161.33^h^	0.11 to 0.28	0.19^h^
	8. At least one of the intervention techniques is linked to theory	19	93.65^h^	0.09 to 0.29	0.19^g^
	3. Intervention based on single theory	12	57.13^h^	0.05 to 0.32	0.18^f^
	10. All theory-relevant constructs are linked to intervention techniques	10	47.70^h^	-0.02 to 0.37	0.18
	11. At least one of the theory-relevant constructs is linked to an intervention technique	18	70.63^h^	0.07 to 0.27	0.17^g^
	7. All intervention techniques are linked to theory	2			

**Behavior Change Technique **					
	35. Stress management	5	6.73	0.27 to 0.72	0.50^h^
	39. General communication skills training	3	4.38	0.25 to 0.73	0.49^h^
	21. Model/demonstrate the behavior	5	24.80^h^	-0.01 to 0.70	0.35^e^
	34. Relapse prevention/coping planning	14	38.31^h^	0.17 to 0.47	0.32^h^
	27. Facilitate social comparison	4	3.25	0.04 to 0.55	0.29^f^
	5. Goal setting (behavior)	25	126.24^h^	0.16 to 0.38	0.27^h^
	7. Action planning	18	101.67^h^	0.13 to 0.37	0.25^h^
	19. Provide feedback on performance	19	77.38^h^	0.09 to 0.34	0.22^g^
	8. Barrier identification/problem solving	26	112.52^h^	0.10 to 0.30	0.20^h^
	20. Provide instruction	25	97.95^h^	0.13 to 0.28	0.20^h^
	22. Teach to use prompts/cues	3	5.45	-0.17 to 0.57	0.20
	4. Provide normative information about others’ behavior	16	94.32^h^	0.07 to 0.28	0.18^g^
	28. Plan social support/social change	15	41.32^h^	0.10 to 0.27	0.18^h^
	13. Provide rewards for behavior	7	7.17	0.09 to 0.28	0.18^h^
	16. Prompt self-monitoring of behavior	28	80.81^h^	0.07 to 0.24	0.16^h^
	1. Provide information on the consequences in general	29	114.14^h^	0.06 to 0.21	0.14^h^
	2. Provide information on the consequences for individual	12	47.57^h^	0.04 to 0.24	0.14^g^
	26. Use of follow up prompts	5	39.35^h^	-0.10 to 0.35	0.13
	17. Prompt self-monitoring of behavioral outcome	13	45.73^h^	-0.03 to 0.26	0.12
	12. Reinforcing effort toward behavior	3	2.89	0.02 to 0.19	0.11^f^
	36. Emotional control training	11	35.39^h^	-0.03 to 0.22	0.09
	3. Provide information about others’ approval	5	10.48^f^	-0.11 to 0.23	0.06
	6. Goal setting (outcome)	2			
	10. Prompt review of behavioral goals	2			
	14. Shaping	2			
	23. Environmental restructuring	2			
	25. Prompt practice	2			
	24. Agree behavioral contract	1			
	31. Fear Arousal	1			
	32. Prompt self-talk	1			
	37. Motivational interviewing	1			
	9. Set graded tasks	0			
	11. Prompt review of outcome goals	0			
	15. Prompting generalisation of behavior	0			
	18. Prompting focus on past success	0			
	29. Prompt identification as role model	0			
	30. Prompt anticipated regret	0			
	33. Prompt use of imagery				
	38. Time management				
	40. Provide non-specific social support				

**Mode of Delivery: Automated Functions **					

	b. Automated tailored feedback	18	83.75^h^	0.07 to 0.28	0.18^g^
	a. Enriched information environment	30	117.24^h^	0.07 to 0.23	0.15^h^
	c. Automated follow-up messages	14	49.81^h^	-0.01 to 0.19	0.09

**Mode of Delivery: Communicative Functions **						
	d. Access to advisor to request advice	23	121.15^h^	0.16 to 0.42	0.29^h^
	e. Scheduled contact with advisor	13	35.70^h^	0.09 to 0.36	0.22^g^
	f. Peer-to-peer access	20	88.21^h^	0.09 to 0.21	0.20^h^

**Mode of Delivery: Additional Modes **					

	i. Text message (SMS)	4	39.22^h^	0.14 to 1.49	0.81^a^
	h. Telephone	7	19.02^g^	0.09 to 0.61	0.35^g^
	g. Email	19	143.98^h^	0.07 to 0.29	0.18^g^
	j. CD-ROM	1			
	k. Videoconferencing	1			

^a^Effect sizes are ordered within category by size of effect. Characteristics supported by less than three interventions were not examined in order to ensure reliable evaluations of the impact of particular intervention characteristics on effect size.

^b^k = the number of interventions included in the estimate of effect size

^c^Q = homogeneity across the subgroup of interventions

^d^d+ = weighted average effect size

^e^Removing Mikolajczak et al [70] from the evaluation of the effects of modeling on behavior change rendered the effect size significant (k = 4, Q = 13.84, 95% CI 0.14 to 0.84, d+ = 0.49,*P *= .006)

^f^ *P *< .05

^g^ *P * < .01

^h^ *P *< .001

## Discussion

### Overall Findings

The primary aim of the present review was to relate the characteristics of Internet-based interventions to their effectiveness in promoting health behavior change. Like previous reviews, the interventions tended to have variable effects on behavior (ie, the homogeneity Q statistic was significant), and the average effect on behavior was statistically small. Thus, while some interventions had very large effects (d > 1.00) on behavior (eg, [21,71,72]), others were found to have small or even negative effects on behavior (eg, [73,74]). The considerable variability in the effectiveness of Internet-based interventions makes it important to systematically identify the characteristics of effective interventions and to relate these to effect size.

### Use of Theory

Interventions differed substantially in their use of theory, but more extensive use of theory was associated with larger effect sizes. This finding is consistent with assertions that interventions can benefit from using behavior change theory [23-27] and extends the evidence base to interventions delivered on the Internet. Three theories–social cognitive theory (SCT) [22], the transtheoretical model (TTM) [60], and the theory of reasoned action/planned behavior (TPB) [20,61]–were used much more frequently than others. However, only the use of the TPB to inform intervention design led to substantially larger effects than were observed across all interventions. Effect sizes were small-to-medium, comparable to those reported in reviews of non-Internet interventions that used the TPB to develop the intervention [75], and were not simply the consequence of TPB interventions targeting a different set of behaviors. (Interventions based on the TPB targeted a similar range of health-related behaviors to those based on the TTM or SCT.) The observed effectiveness of the TPB in promoting health behavior change stands in contrast to recent assertions that the TPB is primarily a predictive model rather than a model of behavior change that can inform interventions (eg, [76]). However, the heterogeneity of effects across findings means that the findings should be treated with caution and should provide an empirical basis for experimental studies that can demonstrate cause and effect [77,78]. Such studies are also important because Michie and Prestwich’s coding of use of theory [14] used in the present review is, necessarily, based on what is reported in the manuscripts; it is of course possible that manuscripts can report having used theory without actually having done so (and vice versa).

### Behavior Change Techniques

The finding that interventions that incorporated more behavior change techniques tended to have larger effects than interventions that incorporated fewer techniques justified the investment in relatively elaborate interventions. This finding may be a consequence of different techniques targeting different aspects of the behavior change process [18], and future research might usefully consider how particular combinations of techniques might be especially effective in promoting behavior change [33]. However, there is also evidence that very simple interventions can prove effective in some contexts (eg, providing instruction for influencing online food purchases [79] and if-then planning for promoting dental flossing [7]), and issues of cost versus benefit should always be a consideration in designing interventions to promote health behavior change [80]. Tate et al [81] provide a useful discussion of cost versus effectiveness in relation to Internet-based interventions, and we echo their call for future research to collect cost-effectiveness data.

The two behavior change techniques that were associated with the greatest changes in behavior were stress management and general communication skills training. It is interesting that both techniques influence behavior change indirectly via mechanisms such as facilitating problem-solving, promoting self-efficacy [82], or diminishing the impact of stressors that may prevent behavior change [83]. However, relatively few interventions employed these techniques, so the findings should be treated with caution and form the basis for future research. Given the effectiveness of stress management training, it is perhaps surprising that emotional control training was less effective in promoting behavior change. Of the 11 interventions (45%) that incorporated emotional control training, 5 reported negative effect sizes on behavior [31,32,84,85]. Authors reported that in many of these interventions they simply included “strategies to manage mood [85]”or “information on ... dealing with relationships and feelings [31].” In contrast, stress management training tended to be more intensive. For example, the intervention reported by Hänggi [86] incorporated 4 stress management modules that were based on cognitive behavioral principles. Again, these differences might form a useful basis for future empirical investigation.

Two other findings in relation to behavior change techniques warrant comment. First, it was notable that providing information about others’ approval (subjective or injunctive norms) seemed to be less effective than providing normative information about others’ behavior (descriptive norms, d_+_= 0.06 and 0.18, respectively). This finding supports the distinction between the two types of normative influence [87] and research that shows that descriptive norms can exert a more powerful effect on behavior and decision making than injunctive norms (eg, [88,89]). Second, effect sizes associated with modeling, while substantial overall, were also highly variable rendering the overall estimate of effectiveness non-significant. Modeling is usually used to boost self-efficacy [22], and the present interventions tended to incorporate embedded videos demonstrating the focal behavior within the online intervention (eg, [70,90,91]). The variability in effect sizes in the present review was primarily caused by Mikolajczak et al’s “Queermasters” intervention [70], which reported a negative effect on uptake of HIV testing at the three month follow up (d = -0.23). The authors attributed this finding to the relatively short follow-up, which may not have given participants opportunity to act on their newly formed positive intentions. Removing Mikolajczak et al from the evaluation of the effects of modeling on behavior change rendered the effect size significant (*k *= 4, Q = 13.84, 95% CI = 0.14-0.84, d_+_= 0.49, *P *= .006).

### Mode of Delivery

The present review developed a novel coding scheme for the mode by which Internet-based interventions are delivered. Dividing mode of delivery into automated functions, communicative functions, and use of supplementary modes proved informative, with distinct effects being identified within each category. Text messages were highly effective and used in several ways: to promote interaction with the intervention [4,5], send motivational messages (eg, reminders of the benefits of exercise [37]), challenge dysfunctional beliefs [71], or provide a cue to action [35]. Use of communicative functions, especially access to an advisor to request advice, also tended to be effective. It may be that, although the Internet provides a suitable medium for delivering interventions, personal contact via email [92], online [93,94], or text message [95] helps to support behavior change.

### Conclusion

The present review is, to our knowledge, the first to systematically code the characteristics of Internet-based interventions designed to promote behavior change and to link these characteristics to effect size. The strengths of the review are the systematic, meta-analytic approach, the use of established coding frames where possible, and the large number of different interventions that focus on a range of different behaviors. The findings suggest that the effectiveness of Internet-based interventions is associated with more extensive use of theory (in particular the use of the theory of planned behavior), inclusion of more behavior change techniques, and use of additional methods of interacting with participants (especially text messages). The review provides a framework for research that can contribute to a science of Internet-based interventions [1] and our findings provide a rationale for investing in more intensive theory-based interventions that incorporate multiple behavior change techniques and modes of delivery. However, the heterogeneity of effects across findings and the relatively small number of interventions associated with some characteristics mean that the findings should be treated with caution and provide an empirical basis for experimental studies that can demonstrate cause and effect.

## Multimedia Appendix 1

Effect Sizes for Interventions Included in the Meta-Analysis

## Multimedia Appendix 2

Intervention Characteristics for Interventions Included in the Meta-Analysis

## Abbreviations

DIM: diffusion of innovations model
ELM: elaboration likelihood model
EPPM: extended parallel process model
HBM: health belief model
PAPM: precaution adoption process model
SCT: social cognitive theory
SMS: short message service
SNT: social norms theory
SRT: self-regulation theory
TPB: theory of reasoned action/planned behavior
TTM: transtheoretical model
