# Supplementary material for: Using the Internet to Promote Health Behavior Change: A Systematic Review and Meta-analysis of the Impact of Theoretical Basis, Use of Behavior Change Techniques, and Mode of Delivery on Efficacy
Source: J Med Internet Res. 2010 Feb 17;12(1):e4. doi: 10.2196/jmir.1376 (PMC2836773; doi:10.2196/jmir.1376)
Supplement: Supplementary file 1 [file jmir_v12i1e4_app1.pdf]

Multimedia Appendix 1. Effect sizes for interventions included in the meta-analysis

Authors	Behavior	N_E	N_C	Effect size *g*
An, Hennrikus, Perry, Lein, Klatt, Farley et al (2007) [96]	Recruitment to interventions	734	1490	-0.20[c]
Bersamin, Paschall et al (2007) [97] - baseline drinkers	Alcohol consumption	60	79	0.50[b]
Bersamin, Paschall et al (2007) [97] - baseline nondrinkers	Alcohol consumption	118	113	-0.01
Bosak, Yates, & Pozehl (2007) [98]	Physical activity	12	9	0.82
Brendryen & Kraft (2008) [4]	Smoking abstinence	197	199	0.35[b]
Brendryen, Drozd, & Kraft (2008) [5]	Smoking abstinence	144	146	0.28
Buller, Woodall, Zimmerman, Slater, Heimendinger et al (2008) [99]	Dietary behavior	242	231	0.15[a]
Carr, Bartee, Dorozynski, Broomfield et al (2008) [21] - Study 1	Physical activity	4	5	1.49[a]
Carr, Bartee, Dorozynski, Broomfield et al (2008) [21] - Study 2	Physical activity	14	18	0.13
Celio, Winzelberg, Wilfley, Eppstein-Herald, Springer et al (2000) [100]	Dietary behavior	24	19	0.31
Chan, Callahan, Hatch-Pigott, Lawless, Proffitt, Manning et al (2007) [92]	Symptom diary submission	60	60	0.29
Chiauzzi, Green, Lord, Thum, & Goldstein (2005) [101]	Alcohol consumption	105	110	0.26
Christakis, Zimmerman, Rivara, & Ebel (2006) [102]	Use of preventive care practices	200	177	0.19[c]
Christensen, Liana, Barney, Mackinnon, & Griffiths (2006) [103]	Help-seeking for depression	102	130	0.09
Cintron, Phillips, & Hamel (2006) [104]	Completion of document	430	482	-0.05
Cook, Billings, Hersch, Back, & Hendrickson (2007) [105]	Physical activity	209	210	0.08

	Dietary behavior			0.11
Cussler, Teixeira, Going, Houtkooper, Metcalfe, Blew et al (2008) [106]	Physical activity	66	69	-0.02
	Dietary behavior			0.12
Etter (2005) [107]	Smoking abstinence	1896	2341	-0.08[a]
Fordis, King, Ballantyne, Jones, Schneider, Spann et al (2005) [108]	Physician performance	17	18	0.54[a]
Frosch, Kaplan, & Felitti (2003) [73]	Service uptake	94	106	-0.52[a]
Glasgow, Boles, McKay, Feil, & Barrera (2003) [93]	Dietary behavior	37	33	0.03
Gold, Burke, Buzzell, Pintauro, & Harvey-Berino (2007) [80]	Dietary behavior	40	48	0.61[b]
Gollings & Paxton (2006) [109]	Dietary behavior	20	19	0.12
Hager, Hardy, & Aldana (2001) [110]	Physical activity	201	201	0.09
Hänggi (2004) [85]	Behavioral responses to stress	41	40	0.62[c]
Heinicke, Paxton, McLean, & Wertheim (2007) [111]	Dietary behavior	28	34	0.52[c]
Huang, Barzi, Huxley, Denyer, Rohrlach, Jayne, & Neal (2006) [79]	Food purchase	246	251	0.55[c]
Hurling, Catt, Boni, Fairley, Hurst, Murray et al (2007) [71]	Physical activity	47	30	2.25[c]
Hurling, Fairley, & Dias (2006) [37]	Physical activity	23	20	0.53
Jacobi, Morris, Beckers, Bronisch-Holtze, Winter et al (2007) [112]	Dietary behavior	47	50	0.31[a]
Jago, Baranowski, Baranowski, Thompson et al (2006) [113] - Wave 1	Physical activity	40	40	0.06
Jago, Baranowski, Baranowski, Thompson et al (2006) [113] - Wave 2	Physical activity	108	108	0.24[b]

Japuntich, Zelmer, Smith, Jorenby, Valdez, Fiore et al (2006) [114]	Smoking abstinence	140	144	0.15
Jones, Luce, Osborne, Taylor, Cunning, Doyle et al (2008) [84]	Dietary behavior	44	43	-0.08
Kim & Kang (2006) [72]	Physical activity	28	23	1.27[c]
Kosma, Cardinal, & McCubbin (2005) [115]	Physical activity	46	29	0.36
Kypri & McAnally (2005) [116]	Physical activity	61	61	0.71[a]
	Dietary behavior			0.65[a]
	Alcohol consumption			-0.11
Lin, Wittevrongel, Moore, Beaty, & Ross (2005) [117]	Patient-provider communication	175	166	0.72[c]
Lorig, Ritter, Laurent, & Plant (2006) [118]	Physical activity	354	426	0.13[a]
	Behavioral responses to stress			0.09[a]
	Patient-provider communication			0.05
Marks, Campbell, Ward, Ribisl, Wildemuth, & Symons (2006) [34]	Physical activity	158	161	-0.43[b]
Marshall, Leslie, Bauman, Marcus, & Owen (2003) [119]	Physical activity	250	262	-0.03
McKay, Danaher, Seeley, Lichtenstein, & Gau (2008) [85]	Smoking abstinence	1159	1159	-0.04
McKay, King, Eakin, Seeley, & Glasgow (2001) [120]	Physical activity	35	33	-0.14
Meigs, Cagliero, Dubey, Murphy-Sheehy, Gildesgame et al (2003) [121]	Service uptake	307	291	0.01
Mevissen, Ruiter, Meertens, Zimbile, & Schaalma (Forthcoming) [90]	Sexual behaviors	33	37	0.12
Mikolajczak, Van Breukelen, Kok, & Hospers (2008) [70]	Service uptake	242	287	-0.23

Moore, Soderquist, & Werch (2005) [35]	Alcohol consumption	53	47	0.13
Munoz, Lenert, Delucchi, Stoddard, Perez et al (2006) [32] - Study 3	Smoking abstinence	141	139	-0.43[a]
Munoz, Lenert, Delucchi, Stoddard, Perez et al (2006) [32] - Study 4	Smoking abstinence	142	146	-0.07
Napolitano, Fotheringham, Tate, Sciamanna, Leslie et al (2003) [122]	Physical activity	21	31	0.37
Nguyen, Donesky-Cuenco, Wolpin, Reinke, Benditt et al (2008) [123]	Physical activity	19	20	-0.15
Oenema, Brug, Dijkstra, de Weerdt, & de Vries (2008) [124]	Physical activity	887	930	-0.01
	Dietary behavior			0.16[b]
	Smoking abstinence			0.18
Paschall, Bersamin, Fearnow-Kenney, Wyrick, & Currey (2006) [125]	Alcohol consumption	173	197	0.16[b]
Patten, Croghan, Meis, Decker, Pingree, Colligan et al (2006) [31]	Smoking abstinence	70	69	-0.47
Pike, Rabius, McAlister, & Geiger (2007) [126]	Smoking abstinence	5404	1047	0.01
Prestwich (2003) [127] - approach behaviors	Health behaviors	467	240	-0.01
Prestwich (2003) [127] - avoidance behaviors	Health behaviors	120	53	0.00
Prochaska, Butterworth, Redding, Burden, Perrin, Leo et al (2008) [128]	Physical activity	503	464	0.23[b]
	Behavioral responses to stress			0.31[c]
	Smoking abstinence			0.16
Riper, Kramer, Smit, Conijn, Schippers, & Cuijpers (2008) [129]	Alcohol consumption	130	131	0.58[c]
Ritterband, Cox, Walker, Kovatchev, McKnight, Patel et al (2003) [130]	Pediatric encopresis	12	12	0.80[c]

Roberto, Zimmerman, Carlyle, & Abner (2007) [131]	Sexual behaviors	125	183	0.51[a]
Rodriguez, von Glahn, Rogers, Chang, Fanjiang, & Safran (2006) [74]	Survey response rate	1477	2362	-0.84[c]
Ross, Moore, Earnest, Wittevrongel, & Lin (2004) [132]	Medication / general adherence	38	43	0.46[a]
Saitz, Palfai, Freedner, Winter, MacDonald, Lu et al (2007) [133]	Alcohol consumption	126	109	-0.09
Shimazu, Kawakami, Irimajiri, Sakamoto, & Amano (2005) [134]	Stress-related problem solving	100	104	0.13
Skår, Sniehotta, Molloy et al (in press) [6]	Physical activity	335	315	0.10
Sniehotta, Araújo-Soares, & Dombrowski (2007) [7]	Dental flossing	64	70	0.32
Spittaels, De Bourdeaudhuij, & Vandelanotte (2007) [135]	Physical activity	103	104	0.32[c]
Spittaels, De Bourdeaudhuij, Brug, & Vandelanotte (2007) [19]	Physical activity	116	141	0.07
Steele, Mummery, & Dwyer (2007) [136]	Physical activity	48	49	-0.30
Strecher, Shiffman, & West (2005) [137]	Smoking abstinence	1759	1742	0.16[b]
Strecher, McClure, Alexander, Chakraborty, Nair et al (2008) [138]	Smoking abstinence	488	456	0.20[a]
Swartz, Noell, Schroeder, & Ary (2006) [139]	Smoking abstinence	171	180	0.54[a]
Thombs, Olds, Osborn, Casseday, Glavin, & Berkowitz (2007) [140]	Alcohol consumption	192	192	-0.02
Tomnay, Pitts, Kuo, & Fairley (2006) [141]	Contact tracing for STD	68	29	-0.18
van den Berg, Ronday, Peeters, le Cessie et al (2006) [142]	Physical activity	77	75	0.59[c]
Vinokur, Merion, Couper, Jones, & Dong (2006) [143]	Service uptake	331	159	0.22
Wade, Carey, & Wolfe (2006) [91]	Child behavior problems	19	20	0.61[c]

Walters, Vader, & Harris (2007) [144]	Alcohol consumption	41	41	-0.22
White, Martin, Newton, Walden, York-Crowe et al (2004) [145]	Physical activity	23	27	0.32
	Dietary behavior			0.18
Williamson, Walden, White et al (2006) [49] – child sample	Physical activity	23	28	0.36
	Dietary behavior			0.00
Williamson, Walden, White et al (2006) [49] – adult sample	Physical activity	26	26	0.11
	Dietary behavior			-0.12
Winett, Anderson, Wojcik, Winett, & Bowden (2007) [146]	Physical activity	310	291	0.16
	Dietary behavior			0.14^{b}
Zabinski, Pung, Wilfley, Eppstein, Winzelberg et al (2001) [147]	Dietary behavior	27	29	-0.43^{a}
Zabinski, Wilfley, Calfas, Winzelberg, & Taylor (2004) [148]	Dietary behavior	30	30	0.77^{b}

Note. g = standardized mean difference with Hedge's adjustment. N_E = number of participants in the experimental group, N_C = number of participants in the control group. ^{a} $P < .05$, ^{b} $P < .01$, ^{c} $P < .001$.
