# Supplementary material for: Using the Internet to Promote Health Behavior Change: A Systematic Review and Meta-analysis of the Impact of Theoretical Basis, Use of Behavior Change Techniques, and Mode of Delivery on Efficacy
Source: J Med Internet Res. 2010 Feb 17;12(1):e4. doi: 10.2196/jmir.1376 (PMC2836773; doi:10.2196/jmir.1376)
Supplement: Supplementary file 2 [file jmir_v12i1e4_app2.pdf]

Multimedia Appendix 2. Intervention characteristics for interventions included in the meta-analysis

Study	Theoretical. basis	Use of theory	Behavior change techniques	Mode of delivery
An et al (2007) [96]	none reported	none reported	26	c, g
Bersamin et al (2007) [97] – drinkers	TPB	1, 5, 9, 11	1, 4, 16, 19	a, b, f
Bersamin et al (2007) [97] – nondrinkers	TPB	1, 5, 9, 11	1, 4, 16, 19	a, b, f
Bosak et al (2007) [98]	SCT	1, 3, 5	none reported	none reported
Brendryen & Kraft (2008) [4]	none reported	none reported	26, 34	b, d, i
Brendryen et al (2008) [5]	SCT, SRT	1, 2, 5, 8, 11	7, 16, 26, 34, 35, 36	b, c, g, h, i
Buller et al (2008) [99]	DIM, SCT	1, 5, 9, 11	1, 3, 4, 5, 20	c
Carr et al (2008) [21] - Study 1	SCT, TTM	1, 5, 6, 8, 9, 10, 11	5, 7, 8, 13, 16, 27, 28, 34, 35	e, h
Carr et al (2008) [21] - Study 2	SCT, TTM	1, 5, 6, 8, 9, 10, 11	5, 7, 8, 13, 16, 27, 28, 34, 35	e, h
Celio et al (2000) [100]	none reported	2, 5, 8, 10, 11	1, 16, 19, 27, 28	d, f
Chan et al (2007) [92]	none reported	none reported	none reported	a, b, d, e, h
Chiauzzi et al (2005) [101]	none reported	none reported	1, 2, 4, 20, 27, 39	d
Christakis et al (2006) [102]	none reported	none reported	1, 20	none reported
Christensen et al (2006) [103]	none reported	none reported	8, 17, 36	e, h

Cintron et al (2006) [104]	none reported	none reported	1, 20	c, g
Cook et al (2007) [105]	SCT, TTM	1, 5, 8, 11	1, 2, 8, 12, 16, 20, 28, 37	a
Cussler et al (2008) [106]	none reported	none reported	1, 6, 16, 17, 19	a, d, f
Etter (2005) [107]	TPB, TTM	1, 5, 6, 8, 11	1	none reported
Fordis et al (2005) [108]	none reported	2, 5	1, 19, 21, 23	a, d
Frosch et al (2003) [73]	Same as comparison	Same as comparison	Same as comparison	none reported
Glasgow et al (2003) [93]	none reported	2, 5, 8, 10, 11	5, 8, 16, 17, 19, 20	a, d, e
Gold et al (2007) [81]	none reported	none reported	17, 19, 20	d, e
Gollings & Paxton (2006) [109]	none reported	none reported	Same as comparison	e, f
Hager et al (2001) [110]	TTM	1, 3, 5, 6	none reported	none reported
Hänggi (2004) [86]	none reported	none reported	17, 35, 39	d, f
Heinicke et al (2007) [111]	none reported	2, 4, 5, 8, 10, 11	2, 16, 34, 36	d, f
Huang et al (2006) [79]	none reported	none reported	20	b
Hurling, Catt et al (2007) [71]	ELM, TPB	1, 5	4, 5, 7, 8, 19	g, i
Hurling, Fairley, & Dias (2006) [37]	ELM, SCT, TPB, TTM	1, 2, 5	4, 8, 22	c, i
Jacobi et al (2007) [112]	none reported	none reported	none reported	none reported

Jago et al (2006) [113] - Wave 1	none reported	none reported	1, 3, 5, 8, 10, 13, 14, 19, 20	a, b, d
Jago et al (2006) [113] - Wave 2	none reported	none reported	1, 3, 5, 8, 10, 13, 14, 19, 20	a, b, d
Japuntich et al (2006) [114]	none reported	none reported	1, 5, 8, 17, 20, 34, 36	a, c, d, f, h
Jones et al (2008) [84]	none reported	none reported	5, 8, 16, 20, 28, 34, 36	c, d, f,
Kim & Kang (2006) [72]	TTM	1, 3, 5, 6	1, 5, 7, 16, 20	a, d, e, h
Kosma et al (2005) [115]	TTM	1, 2, 3, 4, 5, 8, 9	5, 8, 13, 16, 22, 23, 28	c, g
Kypri & McAnally (2005) [116]	none reported	none reported	4, 16, 19	b
Lin et al (2005) [117]	none reported	none reported	none reported	d
Lorig et al (2006) [118]	none reported	2, 5	7, 20, 28, 32, 36	e, f, g
Marks et al (2006) [34]	Same as comparison	Same as comparison	Same as comparison	a
Marshall et al (2003) [119]	Same as comparison	Same as comparison	Same as comparison	a, g
McKay, Danaher et al (2008) [85]	none reported	1, 3	1, 5, 7, 8, 22, 36	a, d, f, g
McKay, King et al (2001) [120]	none reported	none reported	2, 5, 7, 8, 12, 16, 19, 20	d, f
Meigs et al (2003) [121]	none reported	none reported	none reported	b
Mevissen et al (Forthcoming) [90]	none reported	none reported	1, 2, 4, 5, 8, 21	a
Mikolajczak et al (2008) [70]	SCT	1, 2, 3, 5, 7, 10	1, 3, 21	a

Moore et al (2005) [35]	Same as comparison	Same as comparison	Same as comparison	none reported
Munoz et al (2006) [32] - Study 3	none reported	none reported	16, 17, 20, 36	none reported
Munoz et al (2006) [32] - Study 4	none reported	none reported	16, 17, 20, 36	none reported
Napolitano et al (2003) [122]	SCT	1, 3, 5, 6	1, 5, 7, 8, 13, 16, 17, 28, 34	c, g
Nguyen et al (2008) [123]	Same as comparison	Same as comparison	Same as comparison	none reported
Oenema et al (2008) [124]	PAPM	1, 2, 5, 6, 8, 11	2, 4, 5, 7, 16, 19, 20	b
Paschall et al (2006) [125]	none reported	none reported	2, 4, 16, 20	a, c, f, g
Patten et al (2006) [31]	SCT	1, 5, 8, 11	1, 3, 8, 28, 34, 36	a, d, f
Pike et al (2007) [126]	none reported	none reported	none reported	none reported
Prestwich (2003) [127] – approach	none reported	5	7	none reported
Prestwich (2003) [127] – avoidance	none reported	5	7	none reported
Prochaska et al (2008) [128]	TTM	1, 2, 3, 4, 5, 6	1, 4, 16, 19	b
Riper et al (2008) [129]	none reported	2, 5, 8, 10	5, 34	f
Ritterband et al (2003) [130]	none reported	none reported	7, 20, 21, 34	a
Roberto et al (2007) [131]	EPPM, TPB	1, 2, 5, 8, 11	1, 2, 20	a, j
Rodriguez et al (2006) [74]	none reported	none reported	Same as comparison	none reported

Ross et al (2004) [132]	none reported	none reported	16, 17	d
Saitz et al (2007) [133]	none reported	none reported	1, 2, 4, 17, 31	b
Shimazu et al (2005) [134]	SCT	1, 3, 5, 8, 11	1, 8, 26, 34	a, c
Skår et al (In press) [6]	none reported	1, 5	7	none reported
Sniehotta et al [7]	none reported	1, 5	7	none reported
Spittaels et al (2007) [135]	TPB, TTM	1, 5, 6, 8, 11	2, 4, 7, 8, 20, 28	c, g
Spittaels et al (2007) [19]	TPB, TTM	1, 3, 5, 6, 8, 11	2, 5, 8, 20, 28	a, b, f, g
Steele et al (2007) [136]	Same as comparison	Same as comparison	Same as comparison	c, g
Strecher, Shiffman et al (2005) [137]	none reported	none reported	4, 26, 28	c, g
Strecher, McClure et al (2008) [138]	HBM, SCT, TTM	1, 2, 5, 6, 8, 10	none reported	none reported
Swartz et al (2006) [139]	none reported	none reported	1, 5, 7, 8, 20, 28	a
Thombs et al (2007) [140]	SNT	1, 2, 3, 5, 8, 10	1, 4, 16	a, d
Tomnay et al (2006) [141]	none reported	none reported	1	none reported
van den Berg et al (2006) [142]	none reported	none reported	5, 16, 20	e, f, g
Vinokur et al (2006) [143]	TPB	1, 2, 3, 5, 8, 11	1, 28	a
Wade et al (2006) [91]	none reported	none reported	5, 6, 8, 21, 25, 28, 35, 36, 39	a, d, k

Walters et al (2007) [144]	none reported	2, 5, 7, 10	2, 4, 16, 19	b
White et al (2004) [145]	none reported	2	5, 8, 16, 19, 24, 34	a, b, e, f, g
Williamson et al (2006) [49] – child	none reported	none reported	5, 8, 16, 17, 19	a, b, e, g
Williamson et al (2006) [49] – adult	none reported	none reported	5, 8, 16, 17, 19	a, b, e, g
Winett et al (2007) [146]	none reported	none reported	5, 7, 12, 13, 19	a
Zabinski et al (2001) [147]	none reported	none reported	none reported	f
Zabinski et al (2004) [148]	none reported	none reported	1, 8, 25, 34	d, f, g

Note. 'Use of theory' includes the use of predictors - constructs that are not explicitly linked to a theory by the authors, but that are targeted for intervention because they predict behavior [14].
